# Indications and Outcomes of a Hybrid Method Combining Laparoscopic and Anterior Approaches for Inguinal Hernia Repair

**DOI:** 10.7759/cureus.27117

**Published:** 2022-07-21

**Authors:** Nao Kakizawa, Shingo Tsujinaka, Yuki Mizusawa, Sawako Tamaki, Ryo Maemoto, Erika Machida, Yuta Muto, Masaaki Saito, Nobuyuki Toyama, Toshiki Rikiyama

**Affiliations:** 1 Surgery, Saitama Medical Center, Jichi Medical University, Saitama, JPN; 2 Gastroenterlogical Surgery, Tohoku Medical and Pharmaceutical University, Sendai, JPN

**Keywords:** recurrent inguinal hernia, inguinal hernia after prostatectomy, inguinal hernia repair, hybrid method, complex inguinal hernia

## Abstract

Introduction

Surgery for complex inguinal hernia (IH) (recurrent IH or IH after radical prostatectomy (RP)) may be difficult because of the presumed scar or adhesion in the retropubic space. A hybrid method combining the laparoscopic and anterior approaches (HLAA) in a bidirectional surgical technique may be an option in complex IH cases.

Methods

Patients at our institution who underwent IH repair for complex IH using HLAA from April 2018 to November 2019 were included. We retrospectively evaluated the patient characteristics, IH diagnosis, intraoperative variables, complications, and hernia recurrence during the follow-up period.

Results

Twenty patients were involved in this study. Seven patients underwent hLAA for recurrent IH, whereas the remaining 13 underwent hLAA for IH after RP. Five patients had bilateral IH, all of whom had IH after RP. The type of IH was lateral in 21 patients, medial in six patients, and lateral and medial in two patients. Hernia repair was performed using a patch alone in two patients and a plug and patch in 18 patients. Seroma or hematoma was observed in five patients, and one patient experienced chronic pain. No hernia recurrence was observed during the median follow-up period of 24 months.

Conclusion

hLAA could facilitate precise diagnosis and intraoperative confirmation of repair for recurrent IH and IH after RP. The intraoperative findings and the cause of recurrence can be easily shared among surgeons in hLAA. Further investigations are necessary to determine the long-term efficacy of hLAA in a larger cohort.

## Introduction

Inguinal hernia (IH) repair may be complicated in patients with recurrent hernia or those with a history of pelvic surgeries. The causes of recurrence, surgical approaches (laparoscopic or anterior repair), postoperative complications, and long-term outcomes of recurrent IH have been discussed in various reports [1-4]. A previous study revealed that the surgical approach (laparoscopic or anterior) or the use of a mesh did not affect recurrence and complication rates [1]. In contrast, laparoscopic repair for recurrent IH has been associated with less postoperative pain [5], reduced wound infection rate [6], and earlier return to work [7]. The guidelines of international hernia societies recommend laparoscopic repair for recurrent IH after anterior repair and anterior repair for recurrent IH after laparoscopic repair [8-10].

Although radical prostatectomy (RP) with open surgery, laparoscopic, or robot-assisted surgery is being increasingly performed for older patients with prostate cancer, concerns regarding the increased incidence of IH after RP have emerged. Laparoscopic repair for IH after RP is reportedly difficult due to extensive scar tissue and adhesions and is associated with the risk of bowel obstruction or bladder injury [7,11-14].

For cases of IH after RP, laparoscopic repair is challenging and requires extensive experience in laparoscopic hernia repair [15-17]. The procedure is associated with a higher rate of morbidities. For example, bowel obstruction occurred in 5.7% of patients who underwent surgical treatment for IH after RP, compared to 2.8% for the transabdominal preperitoneal approach (TAPP) for primary IH. It is made of the presence of severe scar tissue [15]. In addition, severe scar tissue might cause bladder injury because complete retropubic preparation is hindered by rigid retropubic scar tissue [16].

Like complex IH after RP, recurrent IH cases are often observed after anterior repair, where laparoscopic repair is complicated by hard scars and adhesions [8,11] or fibrotic tissue in the retropubic space. It makes it challenging to identify the appropriate plane [12]. Despite the recent increase in laparoscopic approaches for such cases of complex IH, some studies have advocated approaches other than those recommended in the guidelines: the anterior approach for recurrent or oversized IH [18] and diagnostic laparoscopy as a decision tool for re-recurrent IH [13].

Therefore, we have proposed a hybrid method combining the laparoscopic and anterior approaches (hLAA) to repair complex IH, including recurrent IH and IH after RP. To date, only a few case reports have reported the short-term results after hLAA [13,14,19], and evaluation of the long-term efficacy of surgery for complex IH is essential. This study aimed to review the indications and long-term outcomes of hLAA, including hernia recurrence.

## Materials and methods

In 2018, we started to perform IH repair using hLAA for complex IH and planned to undertake research regarding perioperative outcomes. Patients who underwent IH repair surgery using hLAA for complex IH from April 2018 to November 2019 at Saitama Medical Center, Jichi Medical University, were included in this retrospective, single-center study. The Bioethics Committee approved this study for Clinical Research, Saitama Medical Center, Jichi Medical University (S19-097), including patients’ informed consent.

At our center, surgeons selected a tailored approach for complex IH following diagnostic laparoscopy. If the laparoscopic approach was predicted to be technically challenging due to extensive scarring or adhesion, hLAA was indicated accordingly.

**Figure 1:**
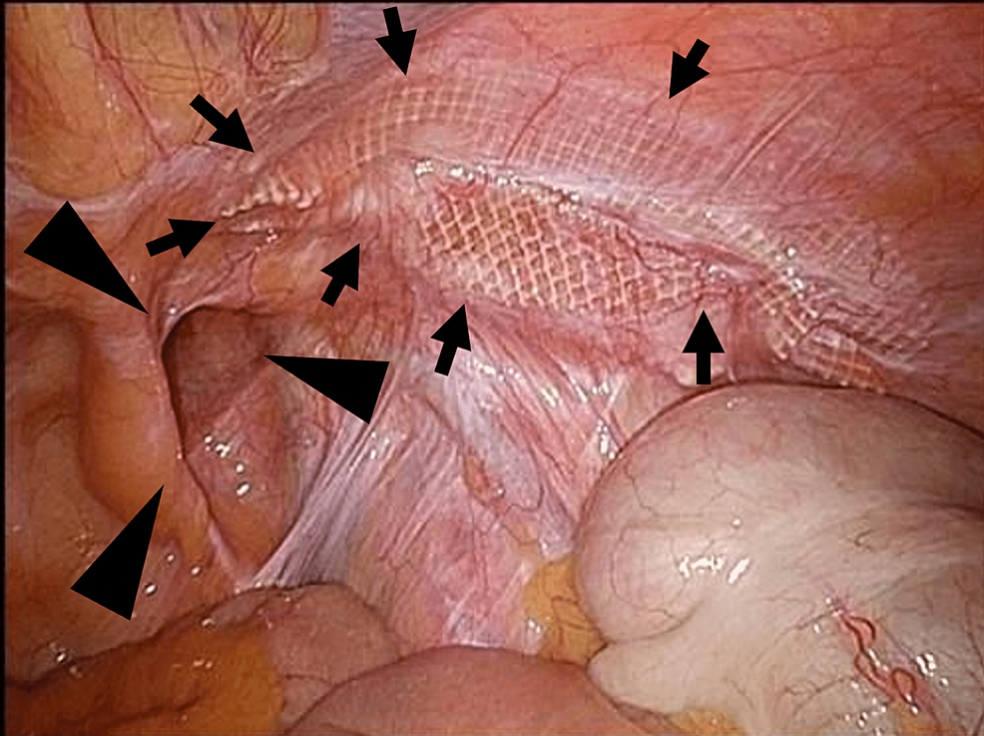
A case of recurrent IH on the right side after TAPP, medial hernia. The mesh (shown by arrows) that was inserted during previous surgery and shrinking showed through the peritoneum, and the hernia orifice (arrowheads) was 3cm in diameter. TAPP- transabdominal preperitoneal approach

**Figure 2:**
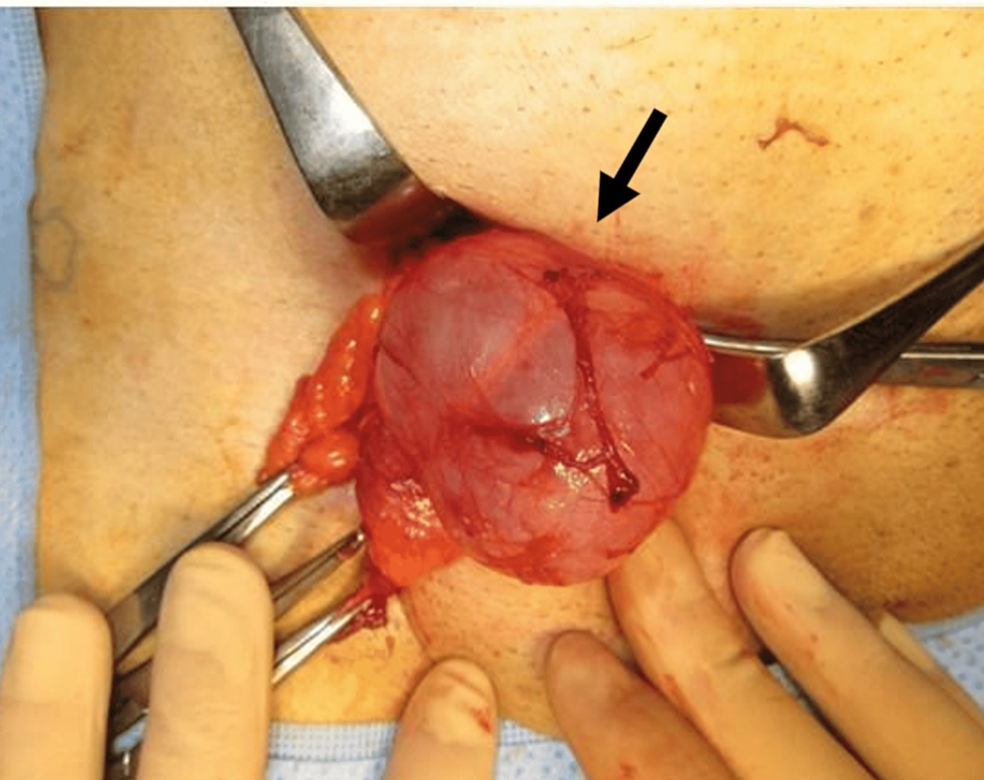
A case of recurrent IH on the right side after TAPP, medial hernia. The hernia sac (arrow) was inflated. TAPP- transabdominal preperitoneal approach

For the laparoscopic approach, we performed TAPP with a conventional, standardized 3-port setting (12mm at the umbilicus and 5mm at the bilateral paraumbilical sites). Initially, the abdominal cavity was insufflated, the type of recurrence was diagnosed, and the hernia orifice was visualized (Figure 1). Then, a 5cm skin incision was made in the inguinal region, and the hernia sac was identified under increased pneumoperitoneum (Figure 2).

**Figure 3:**
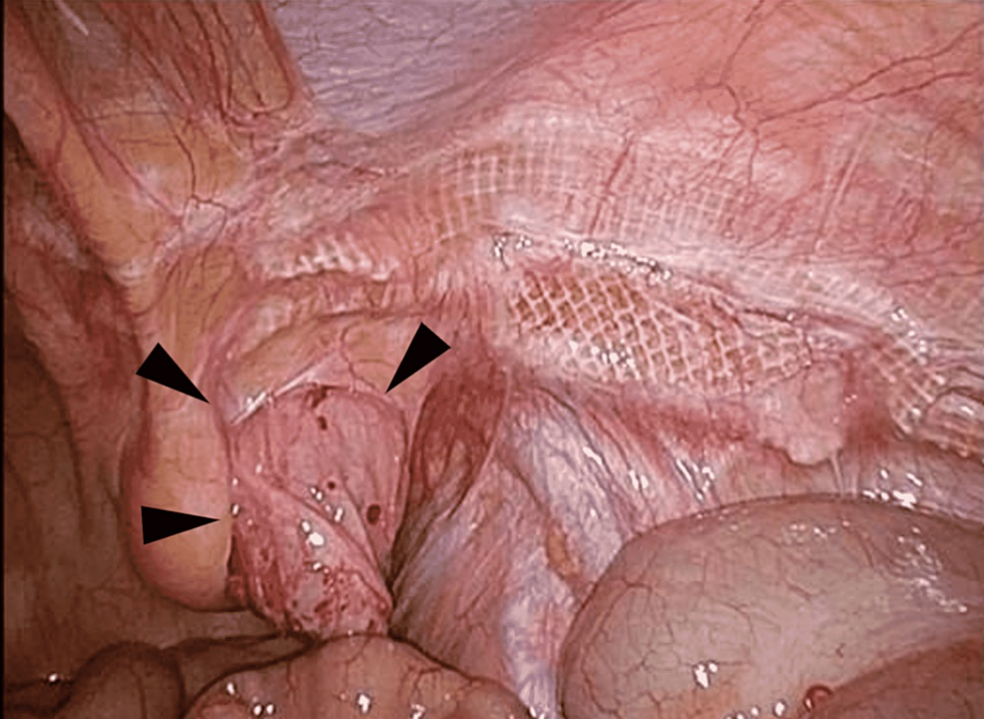
A case of recurrent IH on the right side after TAPP, medial hernia. A repair mesh (arrowheads) was plugged into the hernia orifice. TAPP- transabdominal preperitoneal approach

The tissues surrounding the hernia sac, vessels, nerves, and spermatic cord were isolated, and adequate space in the preperitoneal cavity to insert a plug. The plug and patch were placed and fixed to cover the myopectineal orifice completely. The preexisting mesh was not removed. Finally, coverage of the hernia orifice and mesh placement was confirmed by laparoscopic inspection (Figure 3).

We assessed patient characteristics, including sex, age, American Society of Anesthesiologists Physical Status Classification System (ASA), body mass index (BMI), and intraoperative variables (operative time, estimated blood loss, intraoperative complications), complications after surgery, length of follow-up, and hernia recurrence. Chronic pain was defined as pain that persisted three months after surgery and lasted beyond six months after surgery [20].

Categorical data were reported as numbers and percentages, and continuous variables were reported as medians and ranges. Statistical analyses were performed using the EZR version 2.6-2 software program [18].

## Results

Twenty patients underwent IH repair surgery using hLAA. The patient characteristics are presented in Table 1.

**Table 1:** Patient characteristics IH: Inguinal hernia; BMI: Body mass index; ASA-PS: American Society of Anesthesiologists physical status

Variables		N (%) or Median (range)
Sex	Male	20 (100)
Age (years)		69.5 (56-80)
BMI (kg/m^2^)		24.6 (16.1-28.2)
Indication for IH repair	Recurrent IH	7 (35)
	IH after RP	13 (65)
ASA-PS	1 or 2	16 (80)
	3	4 (20)
Sideness	Bilateral	5 (25)
	Unilateral	15 (75)
	Left	7
	Right	8
Type of hernia	Lateral	21
	Medial	6
	Mixed	2
Antiplatelet or anticoagulant treatment	Yes	3 (15)
	No	17 (85)

All patients were men (median age: 69.5 (range: 56-80) years). The indications for surgery included IH after RP in 13 patients and recurrent IH in seven patients. Two patients in the recurrent IH group had recurrent IH.

The previous repair procedures in the recurrent IH group were TAPP (n=4), bilayer repair (n=2), and trans-inguinal preperitoneal repair (n=1). The location of the IH was unilateral in 15 cases (right: left = 8:7) and bilateral in five patients. All patients with bilateral IH had previously undergone RP, and one had no preoperative diagnosis of bilateral IH. There were five patients with high BMI (over 25.0). Their IH were all unilateral and lateral types, and all were after RP.

**Figure 4:**
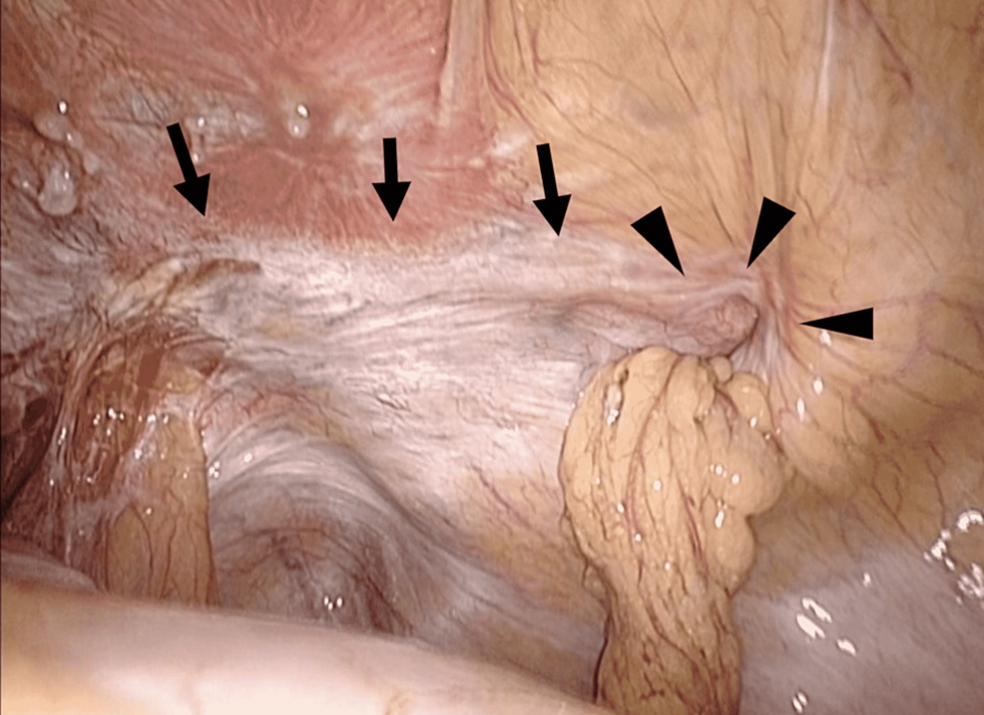
A case of left-sided recurrent IH after bilayer repair, medial hernia. The hernia orifice (arrowheads) was 1.5 cm in diameter and severe adhesion (arrows) was suggested.

**Figure 5:**
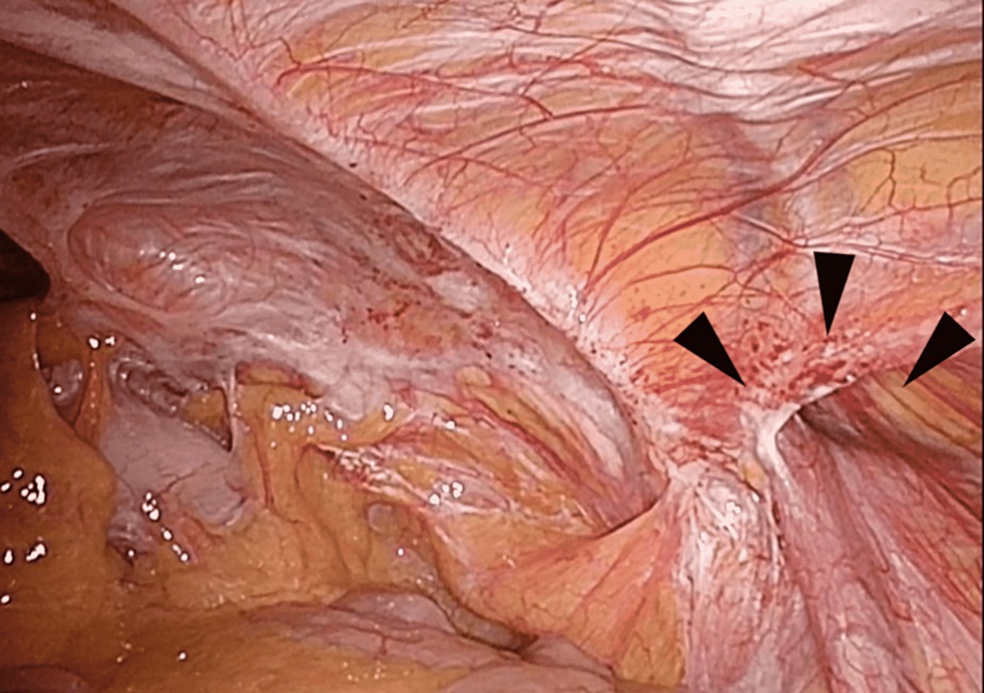
A case of right-sided IH after robot-assisted RP, lateral hernia. The hernia orifice (arrowheads) was 1 cm in diameter.

**Figure 6:**
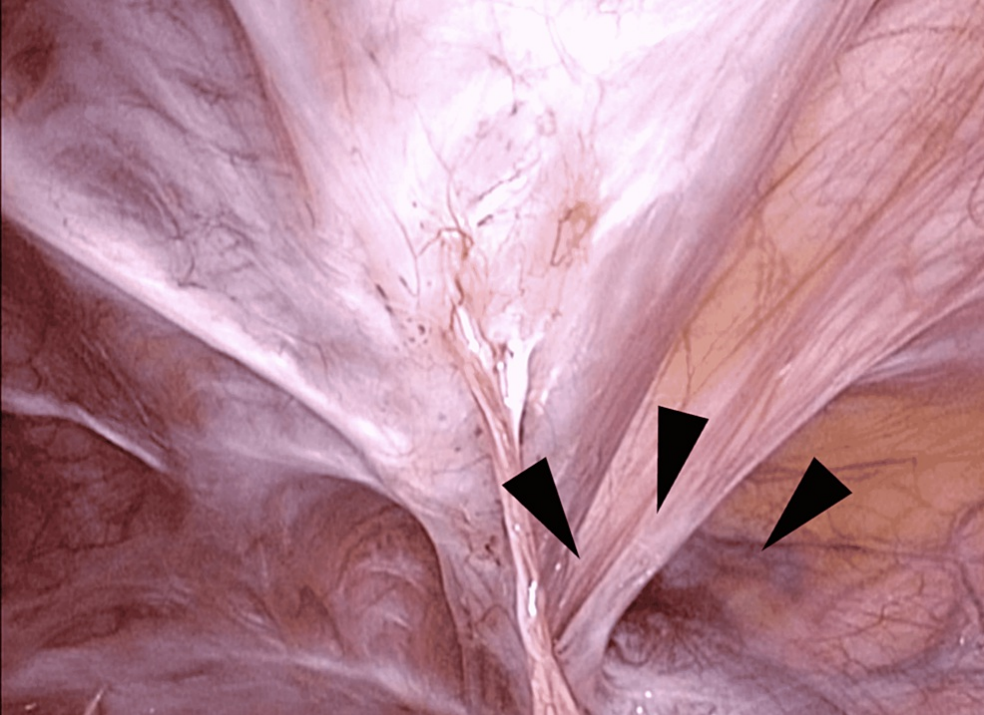
A case of right-sided IH after robot-assisted RP, lateral hernia. The hernia orifice (arrowheads) was 2 cm in diameter.

We documented the surgical difficulties caused by complex IH. Four patients (two with recurrent IH and two with IH after RP) exhibited severe adhesion in the preperitoneal area around the myopectineal orifice at the time of initial laparoscopic exploration. The surgeons decided against continuing laparoscopic surgery and performed hLAA instead (Figures 4, 5, 6).

The type of IH was lateral in 21, medial in six, and both in two patients. All patients with IH after RP showed lateral IH. Hernia repair was performed with a patch alone in two patients and a plug and patch in 18 patients. The median operative time was 125 (range: 77-264) min. The postoperative outcomes are shown in Table 2.

**Table 2:** Postoperative outcomes SSI: Surgical site infection

Variables		N (%) or Median (range)
Blood loss (mL)		10 (0-38)
Operative time (min)		125 (77-264)
Complications	All	6 (30)
	Chronic pain	1 (5.0)
	Seroma or Hematoma	5 (25)
	SSI	0 (0)
	Recurrence	0 (0)
Duration of follow-up (months)		24 (1-42)

Among the patients with recurrent IH, three (15%) experienced pain in the inguinal area after surgery, which required analgesics, including nonsteroidal anti-inflammatory drugs (NSAIDs) for a month or more. One patient with recurrent IH had chronic pain (5.0%) and received NSAIDs for 10 months.

Five patients developed seroma or hematoma that spontaneously resolved within several months. One patient underwent a single-needle aspiration treatment session for hematoma. The median follow-up duration was 24 (range: 1-42) months, and no patient experienced IH recurrence.

## Discussion

In this report, we demonstrated that hLAA for recurrent IH and IH after RP yielded favorable outcomes regarding the precise diagnosis of hernia and intraoperative confirmation of hernia repair. The operative time (median: 125 min) was presumably longer than that of anterior or laparoscopic IH repair alone; however, the incidence of complications after hLAA was similarly low. Further, we showed long-term outcomes of hLAA with no hernia recurrence.

The recurrence rate of IH after laparoscopic repair at the 1-year follow-up was 1.1%, and the recurrence rate of recurrent IH after laparoscopic repair following anterior repair for primary IH was 1.45% [12]. Regarding postoperative complications, many studies and trials have been performed to reduce the complications of recurrent IH, including chronic pain, hematoma, seroma, and re-recurrence [2,3,6,7,11,21,22]. These reports concluded that methods that followed the guidelines provided the best postoperative outcomes and emphasized the importance of expertise because surgery for recurrent IH was primarily tricky. However, some problematic cases of recurrent IH may show higher risks of re-recurrent IH if repaired using only the methods recommended by the guidelines [12].

Many older patients develop IH after RP for prostate cancer. The estimated incidence of IH is 13.7% after open RP, 7.5% after laparoscopic RP, and 7.9% after robot-assisted laparoscopic RP [23]. Intraoperative prophylactic surgical techniques have been proposed to reduce the postoperative incidence of IH [23-25]. However, many complex cases of IH also appear after post-RP anterior IH repair. This could be attributable to severe adhesion in the preperitoneal area, which may impede clear identification of the hernia orifice.

In IH after RP, some patients have bilateral IH [26], highlighting the importance of vigilance to ensure that IH is not missed, which could become an issue for patients in the future. Bilateral IH was not diagnosed preoperatively in one of the five cases of bilateral IH in the present study. hLAA can aid post-RP patients with unilateral IH to prevent future complaints of IH on the other side.

Moreover, in cases involving surgery for recurrent IH, we wish to emphasize the importance of identifying the cause of recurrence, determining the type of IH, and implementing measures to prevent a recurrence. In this regard, hLAA offers several advantages. First, it enables precise and easy hernia classification. Second, it can confirm complete repair of IH by both laparoscopic inspection and direct vision anteriorly, with CO2 insufflation imitating increased abdominal pressure. Third, it is valuable for education and training because the intraoperative findings can be shared among many surgeons. This technique also facilitates discussions about previous surgeries and the complexity of the procedure through video reviews, thereby improving surgical practice in IH repair. In clinical training hospitals, hLAA for complex IH may be helpful in surgical education for residents and attending surgeons.

However, hLAA also has some disadvantages, such as high operative cost, additional operative time, and difficulties in patients with a history of open abdominal surgery with presumptive intra-abdominal adhesions. Moreover, patients with recurrent IH showed postoperative chronic pain, which may be caused by an excessive amount of mesh [27]. Suppose the hernia orifice was sufficiently covered with on-lay mesh under increased pneumoperitoneum. In that case, the patch alone procedure might be better than plug and patch to reduce postoperative chronic pain.

This study also had some limitations. First, this was a retrospective study at a single center involving a small number of patients. Some reports have demonstrated the benefits of laparoscopic repair, even for complex IH [6,7,16]. Therefore, the efficacy of hLAA in comparison with laparoscopic repair for complex IH should require further assessment in a larger cohort. Second, there is a possibility that patients’ characteristics, surgical procedures, and postoperative outcomes differ because of Japan’s large aging population, and IH repair using the anterior approach is still popularly practiced in Japan. We need to consider these factors when we plan and assess subsequent research in a larger cohort.

## Conclusions

In this study, we demonstrated favorable surgical outcomes of hLAA for complex IH, which had the possibility of severe adhesion in the preperitoneal area and difficulty in the usual IH method. Further investigations are required to confirm the long-term efficacy of hLAA and to determine the appropriate patient population for which hLAA should be indicated.
